# Pharmacist Interventions for Prophylactic Antibiotic Use in Urological Inpatients Undergoing Clean or Clean-Contaminated Operations in a Chinese Hospital

**DOI:** 10.1371/journal.pone.0088971

**Published:** 2014-02-25

**Authors:** Hai-Xia Zhang, Xin Li, Hai-Qin Huo, Pei Liang, Jin-Ping Zhang, Wei-Hong Ge

**Affiliations:** 1 Department of Pharmacy, the Affiliated Drum Tower Hospital of Nanjing University Medical School, Nanjing, China; 2 Department of Clinical Pharmacy, School of Pharmacy, Nanjing Medical University, Nanjing, China; Cairo University, Egypt

## Abstract

**Objectives:**

To evaluate the impact and cost-benefit value of pharmacist interventions for prophylactic antibiotic use in surgical patients undergoing clean or clean-contaminated operations.

**Methods:**

A pre-to-post intervention study was performed in the Department of Urological Surgery of a tertiary hospital. Patients admitted from January through June 2011, undergoing clean or clean-contaminated surgery, served as the pre-intervention group; patients admitted from January through June 2012 formed the post-intervention group. Pharmacist interventions were performed for the surgeries in the post-intervention group. The criteria for the rational use of antibiotic prophylaxis were established by the hospital administration. The pharmacist interventions included real-time monitoring of medical records and controlling of the prescriptions of prophylactic antibiotics against the criteria. The pre- and post-intervention groups were then compared to evaluate the outcomes of the pharmacist interventions. A cost-benefit analysis was performed to determine the economic effects of implementing the pharmacist intervention on preoperative antibiotic prophylaxis.

**Results:**

After the pharmacist intervention, a significant decrease was found in the rate of no indications for prophylactic antibiotic use (*p* = 0.004), the rate of broad-spectrum antibiotic use (*p*<0.001), the rate of drug replacement (*p*<0.001) and the rate of prolonged duration of prophylaxis (*p*<0.001). Significant reductions were observed in the mean antibiotic cost (*p*<0.001), the mean duration of antibiotic prophylaxis (*p*<0.001) and the mean number of antibiotics used (*p*<0.001). A significant increase was observed in the rate of correct choice of antibiotics (*p*<0.001). The ratio of the net mean cost savings for antibiotics to the mean cost of pharmacist time was approximately 18.79∶1.

**Conclusion:**

Real-time interventions provided by a clinical pharmacist promoted rational use of prophylactic antibiotics, with a significant reduction in antibiotic costs, thus leading to favorable economic outcomes.

## Introduction

Prophylactic antibiotics are often used by surgeons to prevent surgical site infections (SSIs) following operations [1]. Some guidelines indicate that prophylactic antibiotics appropriately administered before surgical procedures can reduce the incidence of SSIs [1]–[4]; however, inappropriate prescribing and excessive antimicrobial prophylaxis not only increase the risk of adverse effects and promote the emergence of resistant organisms but also increase drug costs and waste healthcare resources [5]. Inappropriate prophylaxis, which is characterized by the unnecessary use of broad-spectrum agents and the continuation of therapy beyond the recommended time period, is common in many countries [6]–[8]. Statistics from the National Health and Family Planning Commission (NHFPC) of China suggest that the most common prophylactic antibiotics for clean or clean-contaminated operations are third-generation cephalosporin and combinations of double beta-lactams [9].

Antibiotic guidelines and associated interventions have been found to be effective in promoting the rational use of antibiotics. Previous studies have demonstrated that pharmacists could play vital roles in antibiotic stewardship programs [10]–[12]. For example, Hand noted that antibiotic specialist pharmacists had become an established feature of the antibiotic stewardship landscape in hospitals throughout the UK [13]. A survey study conducted in the US in 2004 found a negative correlation between onsite pharmacist hours and the use of third-generation cephalosporins and carbapenems [14]. Recently, Shi *et al.* showed that pharmacists in China could promote the rational use of antibiotic prophylaxis in Type 1 incision operations though drug use evaluation [15].

Since 2011, various measures have been taken by the NHFPC to control the irrational use of prophylactic antibiotics in most Chinese urban tertiary hospitals. These measures have four basic components: formulary restriction, surgeon education, feedback activity and guideline development [16]. The interventions were mainly performed by hospital administrators and infection control practitioners. However, in most Chinese hospitals, pharmacists are not involved in controlling irrational use of prophylactic antibiotics [17].

Usually, prophylactic antibiotics are not indicated for clean surgical procedures in urological units. However, if a patient has an underlying medical condition associated with a high risk of post-operative infection, prophylaxis is recommended. These medical conditions include old age, malnutrition, obesity, diabetes, hypoxemia and immunodeficiency [18]. In contrast, some surgical procedures involving the ureters are considered to be clean-contaminated operations. Prophylactic antibiotics should be administered in clean-contaminated procedures or operative procedures with long durations [4], [19].

An audit of prophylactic antibiotic use conducted by hospital administrators in 2011 found that irrational use of antibiotics in the perioperative period of surgical procedures was common in the Department of Urological Surgery of Nanjing Drum Tower Hospital, and included using non-indicated medications, inappropriate choices of antibiotics, improper timing of the administration of the first preoperative dose(s), unnecessarily high doses, long durations and unnecessary replacement of drugs or combinations. Thus, it is necessary to conduct interventions on the use of prophylactic antibiotics for surgical operations in urology. Some studies conducted in US hospitals have shown that the application of real-time pharmacist interventions is an efficient, sensitive and reliable method for evaluating antimicrobial prophylaxis [20].

In this study, to correct the inappropriate use of prophylactic antibiotics and to reduce antibiotic costs, a clinical pharmacist was delegated to monitor the real-time use of prophylactic antibiotics through established criteria for rational use of prophylactic antibiotics in patients undergoing either clean or clean-contaminated operations. The objectives of this study were to evaluate the impact and cost-benefit value of pharmacist interventions for prophylactic antibiotic use.

## Methods

### 2.1 Study design

#### 2.1.1 Study site location and participants

A retro-prospective study was performed on a cohort of patients requiring scheduled clean or clean-contaminated surgery, in which the compliance and appropriateness of the preoperative antimicrobial prophylaxis were evaluated. Data on the characteristics of the surgical patients were collected from the Department of Urological Surgery of Nanjing Drum Tower Hospital, which is a tertiary hospital in the city of Nanjing.

This was a two-stage (pre-/post-intervention) study. The pre-intervention stage without the pharmacist served as an observational period and was performed from January 1, 2011 to June 30, 2011 to determine problems associated with preoperative antibiotic prophylaxis in surgical patients. In the post-intervention stage, which served as an interventional period, a full-time, experienced clinical pharmacist worked in the unit between January 1 and June 30, 2012, to analyze the pharmacist intervention outcomes.

All patients undergoing either clean or clean-contaminated operations in the urological ward were enrolled. To ensure comparability of the statistical data, inclusion and exclusion criteria were established. The inclusion criteria for enrolling patients were: (1) the wound class of the surgical operation was clean or clean-contaminated; and (2) the patients were normal healthy patients, i.e., they only had local pathological changes but no systemic diseases. The exclusion criteria for the medical records were that the patients (1) had undergone any invasive operation within the month prior to surgery; or (2) had received therapeutic antibiotics to treat bacterial infections within the two weeks prior to surgery.

#### 2.1.2 Establishing criteria

With reference to the *Guidelines for Antibacterial Use in Clinical Practice* published in 2004 and the official document for rational use and standard management of antibiotics issued by NHFPC in 2009 [16], [19], the hospital administration established criteria for the rational use of antibiotic prophylaxis during the perioperative period of clean and clean-contaminated operations in the Department of Urological Surgery. The indicators evaluated for rational use of prophylactic antibiotics were assessed against these criteria (Table 1).

**Table 1:** Criteria for rational use of antibiotic prophylaxis in clean and clean-contaminated operations in the Department of Urological Surgery.

Parameter	Justification for rational use
Indications for prophylaxis	Operative range involving the ureters
	Old age (older than 70 years)
	Risk factors for infections, such as diabetes, obesity and malnutrition
	Operative time >3 h
Choice of antibiotic agent	First-generation cephalosporin
	Second-generation cephalosporin
	Ciprofloxacin
	Aztreonam as an alternative for those patients allergic to cephalosporin
Accuracy of the dose	Dose must be based on concentration used for surgical prophylaxis purposes for each antibiotic
Appropriateness of the route	Intravenous injection
Timing of administration of first preoperative dose/s	First preoperative dose/s must be administered within 0.5–1 h before incision
Need for repeated doses during procedure	For procedures lasting more than 3 h
Duration of prophylaxis	Prophylactic antibiotics must be discontinued within 24–48 h after the end of nephrectomy
	Prophylactic antibiotics must be discontinued within 24 h after the end of non-nephrectomy
Need for combination of antibiotics	Indications for anaerobic bacterial pollution, metronidazole 500 mg i.v. can be combined

#### 2.1.3 Pharmacist interventions

**Figure 1:**
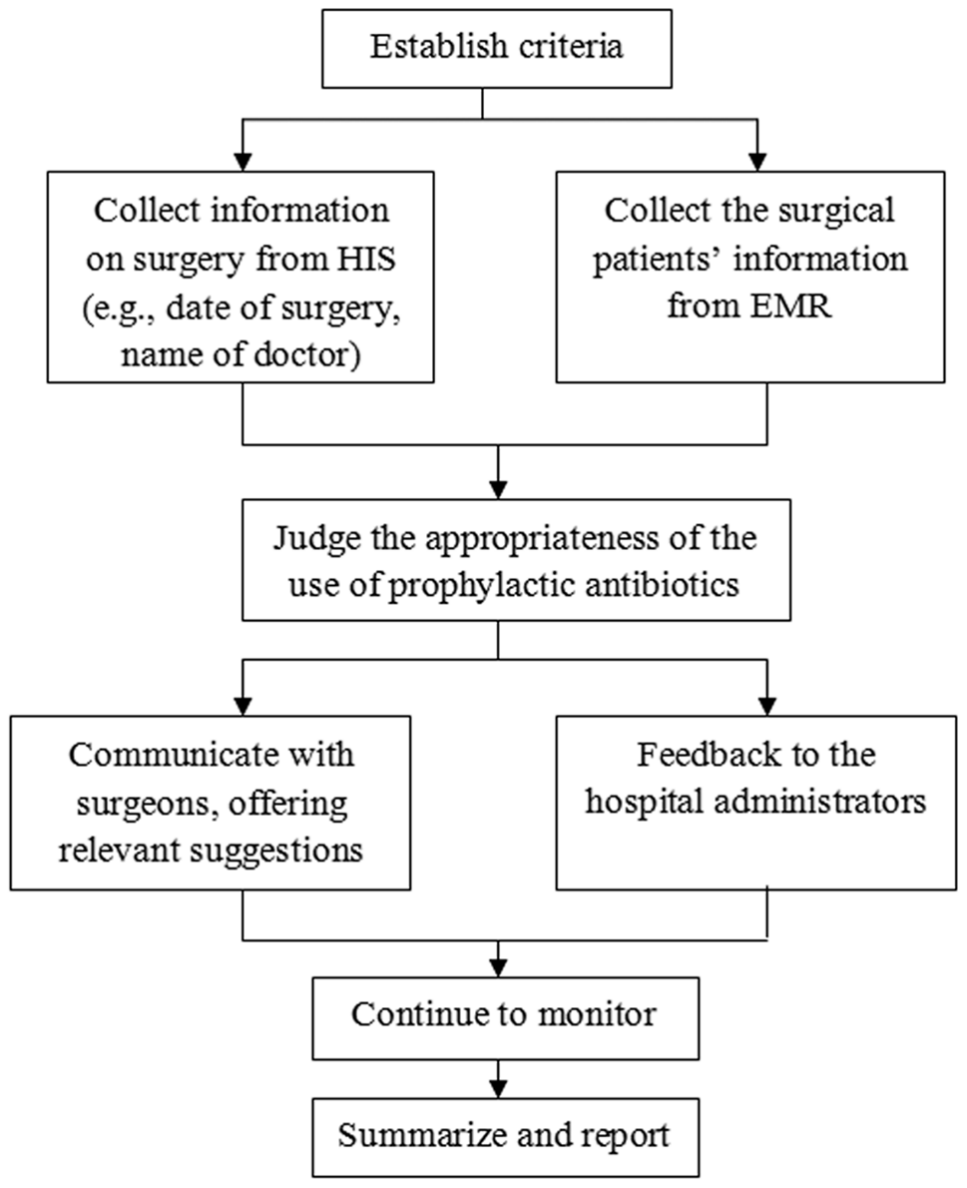
Flowchart for pharmacist intervention. HIS = Hospital Information System; EMR = Electrical Medical Records.

To explore effective intervention measures for prophylactic antibiotics, the interventions provided by the clinical pharmacist were specifically designed for the study in 2012. The pharmacist interventions included real-time monitoring of medical records and controlling the prescription of prophylactic antibiotics against the criteria that were established by the hospital administration. On average, the clinical pharmacist spent 3 hours monitoring electronic medical records (EMR) through the hospital information system (HIS) and communicating with surgeons on every working day during the intervention period. All the real-time monitoring was implemented prior to the surgeries. On the day before the surgery, the clinical pharmacist judged the appropriateness of the use of prophylactic antibiotics by collecting the information for the surgical patient from EMR and information on the surgery from HIS. The pharmacist was responsible for supervising antibiotic administration and controlling noncompliance with the criteria for the rational use of antibiotic prophylaxis. When obviously inappropriate prophylactic antibiotic use was identified, the pharmacist subsequently communicated with the surgeons to correct the medication errors. At the end of each month, the data on irrational use of prophylactic antibiotics were collected and categorized and were reported the hospital administration by the clinical pharmacist. Figure 1 illustrates the workflow of the clinical pharmacist.

### 2.2 Data collection and analysis

All the surgical patients' medical records for the two phases were reviewed by an infection control specialist, who was blinded to the patients' allocation status. A particular Microsoft Excel table (version 2010; Microsoft Corporation, Redmond, WA, USA) was designed to register the patients' demographics (sex, age, diagnosis, allergies, postoperative infections and incision healing), surgical procedures (surgery name and operative time), antibiotic usage (generic names, doses, dosing schedules, timing, duration, combinations and any switchovers to another antibiotic) and cost (total cost of hospitalization, total drug cost and antibacterial drug cost). All costs were recorded in Chinese yuan and were then converted to US dollars (exchange rate, 6.3 yuan  =  US $1). The final values were reported in US dollars.

Cost-benefit analysis, an evaluative technique that entails comparison of the costs of resources consumed in implementing interventions against the benefits resulting from the interventions, was used to determine the economic effects of implementing the pharmacist intervention for preoperative antibiotic prophylaxis. The benefit-to-cost ratio was calculated by dividing the antibiotic cost savings by the cost of the time spent by the clinical pharmacist [21]. The mean antibiotic cost reduction during the post-intervention stage was used to measure the antibiotic cost savings, which were obtained by calculating the difference in mean antibiotic cost between the pre- and post-intervention stages. At the same time, the product of the hourly wage of the clinical pharmacist and the amount of time he spent implementing the interventions was used to measure his cost of time, which was obtained by calculating the mean cost of the pharmacist's time for the total cases during the post-intervention stage. Specifically, it was calculated from the total cost of the pharmacist's time divided by the number of cases during the post-intervention stage.

Data were entered and subsequently analyzed using SPSS software (version 17.0; SPSS, Inc., Chicago, IL, USA). Comparisons between the pre- and post-intervention stages were undertaken in terms of patient characteristics, rate of infection, indications for prophylactic antibiotics, patterns of prophylactic antibiotic usage, antibiotic prophylaxis use, time of administration of the first preoperative dose(s), duration of prophylactic antibiotic use and medical cost per patient. Comparisons between the pre- and post-intervention phases with regard to the age of the patients were conducted using Student's t-test for continuous variables. Categorical variables were expressed as frequencies and percentages. Rates were then analyzed by comparing the proportions using the chi-squared test. The Yates continuity correction was performed when the expected frequencies were <5 [22]–[23]. Trend analysis was performed to evaluate the cost outcomes of patients using simple linear regression analysis [24]. *p*<0.05 was considered statistically significant.

### 2.3 Ethical consideration

The Ethical Committee of Nanjing Medical University approved this retro-prospective study. All patients aged 18 years old or older provided written informed consent for their information to be stored in the hospital database and used for this study. For participants younger than 18 years, we obtained written informed consent from their guardians on their behalf.

## Results

### 3.1 General data of the patients

In total, 370 medical records were included in this study; 196 patients were in the post-intervention group and 174 patients were in the pre-intervention group. General data for the two groups of patients are shown in Table 2. There were no significant differences between the two groups of patients regarding demographic characteristics, such as age, sex, basic medical history, type of surgery and duration of surgery (*p*>0.05). There were no significant differences in postoperative infections between the two groups (*p* = 0.883). Three of 174 cases in the pre-intervention group had lung infections, with an infection rate of 1.72%; three of 196 cases in the post-intervention group had postoperative infections, with an infection rate of 1.53%. Among these three cases, two patients had high fever and suspicion of deep vein infection, and one had a kidney abscess. Thus, these six cases received therapeutic antibiotics after surgery and were excluded from the analysis.

**Table 2:** General data of surgical patients: Pre-intervention versus post-intervention.

Characteristics	Pre-intervention	Post-intervention		*p*-value
	n = 174	n = 196		
Sex				
Male *N* (%)	110 (63.22)	129 (65.82)		NS^a^
Age, Mean ± SD	52.61±14.35	50.43±14.48		NS
Old(>70 years)*N* (%)	12 (6.90)	13 (6.63)		NS
Numbers of patients at high risk for infection *N* (%)	18 (10.34)	18 (9.18)		NS
Type of surgery *N* (%)				
Adrenalectomy	47 (27.01)	50 (25.51)		NS
Renal cyst decortication	47 (27.01)	73 (37.24)		NS
Varicocele ligation	13 (7.47)	18 (9.18)		NS
Nephrectomy	67 (38.51)	55 (28.07)		NS
Operative time *N* (%)				
>3 h	32 (18.39)	41 (20.92)		NS
Postoperative infection rates *N* (%)	3 (1.72)	3 (1.53)		NS

a NS = not significant (*p*>0.05).

### 3.2 Frequency of prophylactic antibiotic usage

As shown in Table 3, in the pre-intervention group, a high rate of ceftizoxime prescription (21.09%) was observed, followed by cefoxitin (18.37%), cefotiam (14.97%), aztreonam (11.9%), levofloxacin (7.48%) and cefamandole ester (7.14%). Cefuroxime (1.70%) was seldom used. In the post-intervention group, a significant decrease was observed in the rate of third-generation cephalosporins used (14.8%). Conversely, the rate of antibiotic prophylaxis using second-generation cephalosporins increased significantly (65.8%) (*p*<0.001). Cefuroxime and aztreonam accounted for 77.01% of the antibiotics used. No significant changes were observed for other antibiotic classes.

**Table 3:** Prophylactic antibiotic use and cost-related characteristics: Pre-intervention versus post-intervention.

	Pre-intervention	Post-intervention	*p*-value
Frequencies of prophylactic antibiotic usage^a^	294	196	-
2nd-generation cephalosporins *N* (%)	126 (42.86)	130 (66.33)	<0.001
Cefuroxime	5 (1.70)	129 (65.82)	-
Cefoxitin	54 (18.37)	1 (0.51)	-
Cefamandole ester	21 (7.14)	0 (0.00)	-
Cefaclor	2 (0.68)	0 (0.00)	-
Cefotiam	44 (14.97)	0 (0.00)	-
3rd- and 4th-generation cephalosporins *N* (%)	106 (36.05)	30 (15.31)	<0.001
Ceftizoxime	62 (21.09)	27 (13.78)	-
Cefodizime	20 (6.80)	2 (1.02)	-
Ceftriaxone tazobactam	17 (5.78)	0 (0.00)	-
Cefoperazone tazobactam	5 (1.70)	0 (0.00)	-
Cefminox	0(0.00)	1(0.51)	-
Cefpirome	2(0.68)	0(0.00)	
Other beta-lactams *N* (%)	35(11.90)	23(11.73)	NS^b^
Aztreonam	35(11.90)	23(11.73)	-
Others^c^ *N* (%)	27(9.18)	13(6.63)	NS
Levofloxacin	22(7.48)	8(4.08)	-
Moxifloxacin	1(0.34)	3(1.53)	-
Clarithromycin	4(1.36)	0(0.00)	-
Azithromycin	0(0.00)	1(0.51)	-
Ornidazole	0(0.0)	1(0.51)	-
Prophylaxis antibiotics indication	171	193	-
Cases required and administered *N* (%)	88(51.46)	80(41.45)	-
Total cases administered *N* (%)	171(100.00)	148(76.68)	<0.001
Cost^d^			
Mean total hospitalization cost (USD)^e^	4141.26	4134.24	NS
Mean total drug cost (USD)^e^	1606.31	1526.17	NS
Mean antibiotics cost (USD)^e^	338.59	98.95	<0.001
Mean duration of antibiotics prophylaxis (days)^e^	7.58	2.91	<0.001
Mean number of antibiotics used^e^	1.73	1.28	<0.001

a There were combinations or replacements of antibiotics in some cases in the two groups, which led the same patient being administered ≥2 types of antibiotics and the frequency of antibiotic usage being higher than the number of patients.

b NS = not significant (*p*>0.05)

c Quinolones, macrolides and nitroimidazoles

d There were no changes in the prices of antibiotics, other drugs or hospital services during the study period.

e Data are expressed as the mean values.

### 3.3 Indications for and rate of prophylactic antibiotic usage

According to the established criteria for the rational use of antibiotic prophylaxis in the perioperative period for renal surgery in the hospital, 80 cases in the post-intervention group and 88 cases in the pre-intervention group showed indications for prophylactic antibiotic usage. However, 171 cases (100%) in the pre-intervention group and 148 cases (76.68%) in the post-intervention group received prophylactic antibiotics. In the post-intervention group, the 80 cases were included among the 148 cases that actually received antibiotics. There was a significant difference in the rate of prophylactic antibiotic usage between the two groups (*p*<0.001) (Table 3).

### 3.4 Medical cost per patient

There were no changes in the price of antibiotics, other drugs or hospital services during the study period. Significant reductions in the mean cost of antibiotics, mean number of antibiotics used and mean duration of antibiotic prophylaxis were observed after the intervention. No significant differences were found in the mean cost of hospitalization or the total drug cost (Table 3).

### 3.5 Inappropriate prophylactic antibiotic use

The rates of inappropriate prophylactic antibiotic use in the pre- and post-intervention groups are summarized in Table 4. (1) Indication: In the pre-intervention group, prophylactic antibiotics were administered in 100% of cases but required in only 51.46% of the cases according to the criteria. Thus, a total of 48.54% of the cases did not require antibiotics but still received them. In contrast, in the post-intervention group, 35.23% of cases received unnecessary prophylactic antibiotics. A significant reduction in unnecessary prophylactic antibiotic use was observed after the intervention (*p* = 0.01). (2) Choice: Significant reductions in the use of unnecessary broad-spectrum and expensive drugs and in the replacement of antibiotics were observed after the intervention (*p*<0.001). Thirteen cases in the pre-intervention group and six in the post-intervention group were inappropriately prescribed combinations of second- or third-generation cephalosporins or aztreonam plus quinolone or macrolide. There were no significant differences in unnecessary combinations between the two groups (*p*>0.05). (3) Dose: In the cases that showed indications for prophylactic antibiotics, all the inappropriate dosing involved unnecessarily high dose of second-generation cephalosporins and aztreonam. There were no significant differences in inappropriate doses between the two groups (*p*>0.05). (4) Timing: Regardless of the choice of antibiotic, only two (2.5%) cases in the post-intervention group were given their first preoperative doses during an inappropriate time frame. (5) Duration of antibiotic prophylaxis: In total, 100% of the cases in the pre-intervention group and 75% of the cases in post-intervention received unnecessarily prolonged prophylaxis. A significant reduction in the rate of unnecessarily prolonged antibiotic prophylaxis was observed between the pre- and post-intervention groups (*p*<0.001).

**Table 4:** Inappropriate prophylactic antibiotic use: Pre-intervention versus post-intervention.

	Pre-intervention	Post-intervention		*p-*value
	*N* (%)	(%) *N*		
Inappropriate decision-making regarding the use of prophylactic antibiotics				
Not required but administered^a^	83 (48.54)	68 (35.23)		0.004
Irrational choice of antibiotic agent^b^				
Unnecessary broad-spectrum and expensive drugs	52 (59.09)	20 (25.00)		<0.001
Unnecessary replacement of drugs	48 (54.55)	22 (27.50)		<0.001
Unnecessary combinations	13 (14.77)	6 (7.50)		NS^c^
Inappropriate dose				
Unnecessarily high doses of second-generation cephalosporins or aztreonam^d^	49 (66.22)	62 (79.49)		NS
Incorrect timing				
Postoperative^b^	0 (0.00)	2 (2.50)		NS
Unnecessary prolonged duration of prophylaxis^b^				
??? >24 h (Non-nephrectomy)	22 (25.00)	30 (37.50)		-
??? >48 h (Nephrectomy)	66 (75.00)	30 (37.50)		-
???+???	88 (100.00)	60 (75.00)		<0.001

a The percentage was calculated by ratio of the number of incorrect cases to the number of total cases that were included in the analysis (171 cases in the pre-intervention group and 193 in the post-intervention group, respectively).

b The calculation range for the percentage only included the number of cases that showed indications for prophylactic antibiotics (88 cases in the pre-intervention group and 80 in the post-intervention group, respectively).

c NS = not significant (*p*>0.05)

d The calculation range for the percentage only included the number of cases that showed indications for prophylactics antibiotic and also received second-generation cephalosporins or aztreonam (74 cases in the pre-intervention group and 78 in the post-intervention group, respectively).

### 3.6 Rate of correct antibiotic administration

Table 5 shows the comparative results for the rate of correct antibiotic administration in the cases that required prophylactic antibiotics in the pre- and post-intervention groups. A total of 22.72% of cases in the pre-intervention group and 68.75% of cases in the post-intervention group complied with the criteria for the choice of antibiotics. A significant increase in the rate of adherence to these criteria was observed after the intervention (*p*<0.001). However, only 6.82% and 7.50% of cases in the pre- and post-intervention groups, respectively, adhered to three of the four criteria. Moreover, none of the 168 cases in the two groups adhered completely to all the criteria.

**Table 5:** Rate of correct antibiotic administration in surgical patients requiring prophylaxis: Pre-intervention versus post-intervention.

		Percentage of correct use of antibiotic				
Antibiotic administration		Pre-intervention (n = 88)		Post-intervention (n = 80)		*p*-value
Correct choice *N* (%)		20 (22.72)	55 (68.75)		<0.001	
Correct choice + correct dose *N* (%)		6 (6.82)	6 (7.50)		NS^a^	
Correct choice + correct dose + correct timing *N* (%)		6 (6.82)	6 (7.50)		NS	
Correct choice + correct dose + correct timing + correct duration *N* (%)		0 (0.00)	0 (0.00)		-	

a NS = not significant (*p*>0.05).

### 3.7 Cost-benefit analysis of pharmacist intervention

In this study, the mean differences in average antibiotic cost for all the cases in the pre- and post-intervention groups were calculated as net mean cost savings for antibiotics. Therefore, the net mean cost savings for antibiotics were $239.64 for the intervention period. With a salary of $6.94 per hour, the total cost of the pharmacist's time was approximately $2,498.40 over 6 months. The mean cost of the pharmacist's time for all the cases in the post-intervention group was calculated as the ratio of the total cost of the pharmacist's time to the total number of cases during the intervention period, yielding an average of $12.75 in human cost. Thus, this calculation resulted in a conservative benefit-to-cost ratio of approximately 18.79∶1 (Table 6).

**Table 6:** Cost-benefit analysis of pharmacist interventions.

Cost of pharmacist time	
Hourly salary	$6.94
Pharmacist time	
3 hours per working day×120 working days during intervention period	360 hours
Total cost of pharmacist time (360 hours×$6.94 per hour)	$2,498.40
Mean cost of pharmacist time (total cost of pharmacist time÷196 cases)	$12.75
Mean antibiotic cost reduction for 193 cases in the post-intervention group	
Mean antibiotic cost for 171 cases in the pre-intervention group-mean antibiotic cost for 193 cases in the post-intervention group	$239.64
Benefit-to-cost ratio	
Mean antibiotic cost reduction for 193 cases: mean cost of pharmacist time	18.79:1

## Discussion

Despite growing concerns regarding the use of prophylactic antibiotics in surgical patients, few data are available on the effectiveness of pharmacist interventions in Chinese hospitals, especially studies reporting the cost-benefit results of introducing a pharmacist as a component of antibiotic stewardship. This study determined the effectiveness of a pharmacist intervention for prophylactic antibiotic use by monitoring prescriptions in real time at the Department of Urological Surgery at Nanjing Drum Tower Hospital.

Although not all surgical patients undergoing clean or clean-contaminated operations require prophylactic antibiotics, 100% of the patients in pre-intervention group received prophylactic antibiotics. However, after the intervention, this number decreased to 76.68% (*p*<0.001). Regardless of the indications for use, the results of this intervention study revealed that usage of broad-spectrum antibiotics, such as third-generation cephalosporins, significantly decreased after the intervention. In contrast, a significant increase in the use of second-generation cephalosporins was found. This increase may have involved the switch from third-generation cephalosporins to second-generation cephalosporins. Based on the data in Table 4, the most common irrational choices of antibiotic agent in the pre-intervention group were as follows: selecting unnecessary broad-spectrum and expensive antibiotics without considering the special spectra of antibiotics and drug prices; unnecessarily high doses of antibiotic agents; unnecessarily frequent replacement of drugs and unnecessary combinations of cephalosporins with other beta lactams or quinolones or macrolides.

Third- or fourth-generation cephalosporins should not be used for SSIs prophylaxis because of their weaker activity against Staphylococcus infections compared to first- or second-generation cephalosporins, the emergence of resistance and high costs [25]. For patients undergoing clean or clean-contaminated operations, prophylactic antibiotic combinations can be used in situations in which certain anaerobic bacteria are not responsive to first- or second-generation cephalosporins. Unless patients have SSIs, all replacements of antibiotics are inappropriate and result in a waste of medical resources or bacterial resistance. During the intervention period, once the pharmacist identified an inappropriate choice of drugs, he communicated to the surgeons that there was little or no additional benefit of a high starting dose for antibiotic agents, replacement of antibiotics or antibiotic combinations. During the first month of the intervention period, the surgeons' rate of resistance to the pharmacist's recommendations reached approximately 50% because of the surgeons' prescribing habits. However, with the pharmacist's continued communications, most of the surgeons gradually recognized the disadvantages of irrational choices of antibiotics and finally accepted the pharmacist's suggestions, which reduced the resistance rate to 5% during the second half of the intervention period. Therefore, the surgeons usually accepted the pharmacist's recommendations during the later stages of the intervention period. During the pharmacist's intervention, the surgeons chose cefuroxime, a relatively inexpensive second-generation cephalosporins in Chinese hospitals, as their first choice. Compared with cefazolin, which is a first-generation cephalosporin, cefuroxime has no advantage regarding pharmacokinetics or price [26]. However, the guidelines published by the Scottish Intercollegiate Guidelines Network (SIGN), which are considered to be the best guidelines with regard to levels of evidence and strength of recommendations [7], indicated that cefuroxime was more effective against Gram-negative bacteria in clinical infections than cefazolin [3]. Most urinary surgeries become infected with Gram-negative bacteria [27]–[30]; therefore, cefuroxime was considered the first choice for prevention of SSIs in this study.

The rate of unnecessary replacement of drugs was found to decrease significantly in the post-intervention group, and the rate of irrational combinations improved. Despite these findings, 75% of cases requiring prophylactic antibiotics in the post-intervention group still received unnecessarily prolonged prophylaxis; the mean duration of antibiotic prophylaxis for all the cases decreased from 7.58 days to 2.91 days. Therefore, it is implied that the supervision of a clinical pharmacist can improve some surgeons' irrational prescribing practices.

For all these reasons, after the intervention, the average cost of antibiotic per patient decreased from a mean value of $338.59 to $98.95 (*p*<0.001). Through the pharmacist intervention, the average cost of antibiotics was significantly reduced, and unnecessary waste of health resources was effectively avoided. The mean cost of the pharmacist's time for all the cases in the post-intervention group was $12.75. The mean cost reduction for all the cases in the post-intervention group was $239.64, which is 18.79 times higher than the mean cost of the pharmacist's time required to implement the intervention. The high cost-benefit ratio found in this study should be presented to hospital administrators and health policy makers to extend this intervention to other surgical departments or hospitals because it indicates that pharmacist interventions in prophylactic antibiotic stewardship are likely to provide favorable economic outcomes.

The timing of the administration of the first dose is an important parameter for rational use of prophylactic antibiotics. In the cases that required prophylactic antibiotics in the present study, the overwhelming majority of patients (166; 98.8%) were administered their first dose during an appropriate time frame. Before implementing this pharmacist intervention, the hospital administrators had undertaken a series of measures to educate surgeons in the prescribing of prophylactic antibiotics. The results showed that the surgeons in the Department of Urology were aware of the optimal timing of dosing and its role in SSI prevention, and they were willing to strictly comply with these criteria.

Although many favorable clinical and economic outcomes were observed in the post-intervention group, it is worth noting that some inappropriate use of prophylactic antibiotics did not improve. For example, 66.22% and 79.49% of the cases in the pre- and post-intervention groups, respectively, received unnecessarily high doses of second-generation cephalosporins and aztreonam. Only 25% of the cases in the post-intervention group complied with the criteria for appropriate duration of prophylactic antibiotics. High-dose or prolonged antibiotic prophylaxis is, at best, of no benefit and, at worst, potentially harmful to patients because of toxicity, the risk of super-infection and the risk of inducing greater bacterial resistance [12], [26]. Therefore, as shown in Table 5, no cases met all the criteria, either in pre- or post-intervention group.

Although the rate of resistance to the pharmacist intervention decreased to a relatively low level by the later stages of the intervention period, the surgeons were not able to completely accept the clinical pharmacist's advice regarding prophylactic antibiotic use. Thus, it was difficult for the clinical pharmacist to correct the inappropriate dosing and duration of antibiotic prophylaxis. The impact of pharmacist interventions could be weakened by other influences that encourage the excessive use of antibiotics. Several factors could be proposed to explain this finding. First, as described by Zhang and Harvey, some misunderstandings regarding antibiotic use are perpetuated among surgeons in China, such as “a longer duration of prophylaxis results in a lower the rate of post-surgical infection” and “new antibiotics are stronger” [31]. Second, the tense doctor-patient relationship in China forces surgeons to protect themselves from lawsuits by practicing defensive medicine, which encourages the prescribing of additional antibiotics to minimize the possibility of infection. In contrast, there is a weak correlation between the timing of the administration of first preoperative dose/s and surgeons' misconceptions regarding antibiotic use, which may be why it is easier to promote the rational timing of administration. Considering the cost-benefit value of pharmacist intervention in the present study, hospital administrators should prolong working time of clinical pharmacists to increase frequency of communication between clinical pharmacists and surgeons, which can help to correct surgeons' misunderstandings. Alternatively, hospital administrators can choose to increase numbers of clinical pharmacists to realize the goal of intervention. Moreover, to maximize the effectiveness of pharmacist interventions and to improve the rational use of clinical antibiotics, clinical pharmacists should be granted the authority to deny inappropriate antibiotic prescriptions.

Several limitations associated with this study should be noted. This intervention study was performed on the basis of a pre-to-post design, in which there was no simultaneous control group. This retro-prospective study was therefore less convincing than a prospective, controlled study design [32]. Additionally, the favorable results obtained cannot be attributed solely to the pharmacist intervention; therefore, a larger sample size and more rigorous design should be employed to evaluate this promising intervention.

## Conclusion

This study demonstrated that real-time interventions provided by a clinical pharmacist could promote the rational use of prophylactic antibiotics, resulting in favorable economic outcomes. These interventions produced substantial antibiotic cost savings over the 6-month study period. The cost savings for each case was 18.79 times the actual pharmacist cost for implementing the intervention. Although the pharmacist intervention had little effect on decreasing inappropriate doses of prophylactic antibiotics, it significantly reduced inappropriate decision-making regarding the use of prophylactic antibiotics, irrational choices of antibiotic agents and unnecessarily prolonged durations of prophylaxis. Based on the cost-benefit analysis in the present study, hospital administrators should prolong working time of clinical pharmacists or increase numbers of clinical pharmacists to monitor prophylactic antibiotics.
